# Supplementary material for: FastqPuri: high-performance preprocessing of RNA-seq data
Source: BMC Bioinformatics. 2019 May 3;20:226. doi: 10.1186/s12859-019-2799-0 (PMC6500068; doi:10.1186/s12859-019-2799-0)
Supplement: Supplementary file 2 — Archive of FastqPuri. Archive containing all files needed to install and run FastqPuri v1.0.6. Date stamp March 22, 2019. (GZ 47,819 kb) [file 12859_2019_2799_MOESM2_ESM.gz › FastqPuri-1.0.6/html/struct__bfkmer.html]

FastqPuri: \_bfkmer Struct Reference


FastqPuri

Public Attributes |
List of all members

\_bfkmer Struct Reference

stores a processed kmer (2 bits pro nucleotide)
More...

`#include <bloom.h>`

|  |  |
| --- | --- |
| Public Attributes | |
| int | kmersize |
|  | |
| int | hashNum |
|  | |
| int | kmersizeBytes |
|  | |
| int | halfsizeBytes |
|  | |
| int | hangingBases |
|  | |
| int | hasOverhead |
|  | |
| unsigned char \* | compact |
|  | |
| uint64\_t \* | hashValues |
|  | |

## Detailed Description

stores a processed kmer (2 bits pro nucleotide)

## Member Data Documentation

## ◆ compact

unsigned char\* \_bfkmer::compact

encoded compactified sequence

## ◆ halfsizeBytes

int \_bfkmer::halfsizeBytes

half size in bytes(needed to decide whether to store a kmer or its reverse complement)

## ◆ hangingBases

int \_bfkmer::hangingBases

number of hanging bases that don't complete a byte

## ◆ hashNum

int \_bfkmer::hashNum

number of hash functions used to construct the filter

## ◆ hashValues

uint64\_t\* \_bfkmer::hashValues

Values of the hash functions

## ◆ hasOverhead

int \_bfkmer::hasOverhead

kmer has overhead when kmersize % 4!=0

## ◆ kmersize

int \_bfkmer::kmersize

kmer size (number of elements)

## ◆ kmersizeBytes

int \_bfkmer::kmersizeBytes

Bytes needed to store the kmer (4bases ~ 1byte)

---

The documentation for this struct was generated from the following file:

- include/bloom.h


---

Generated on Mon Mar 19 2018 23:42:01 for FastqPuri by  

 1.8.14
